# Minireview: The Epigenetic Modulation of *KISS1* in Reproduction and Cancer

**DOI:** 10.3390/ijerph16142607

**Published:** 2019-07-22

**Authors:** Maria Letizia Motti, Rosaria Meccariello

**Affiliations:** Dipartimento di Scienze Motorie e del Benessere, Università di Napoli Parthenope, via Medina 40, 80133 Napoli, Italy

**Keywords:** epigenetics, cancer, reproduction, *KISS1*, *KISS1R*

## Abstract

Epigenetics describes how both lifestyle and environment may affect human health through the modulation of genome functions and without any change to the DNA nucleotide sequence. The discovery of several epigenetic mechanisms and the possibility to deliver epigenetic marks in cells, gametes, and biological fluids has opened up new perspectives in the prevention, diagnosis, and treatment of human diseases. In this respect, the depth of knowledge of epigenetic mechanisms is fundamental to preserving health status and to developing targeted interventions. In this minireview, we summarize the epigenetic modulation of the *KISS1* gene in order to provide an example of epigenetic regulation in health and disease.

## 1. Introduction

Epigenetics describes how lifestyle and environment modulate genome functions without any change in the DNA nucleotide sequence [1], thus affecting human health. The discovery of several epigenetic mechanisms (i.e., DNA methylation of CpG islands within gene promoters, chromatin remodeling, production of non-coding RNA) [2,3,4,5,6] and the possibility of delivering epigenetic marks (i.e., non-coding RNA) in biological fluids or target cells via exosomes or microvesicles has opened up new perspectives in the prevention, diagnosis, and treatment of human diseases [7,8,9,10,11,12].

Nevertheless, insights from genome-wide studies demonstrate that the epigenetic signature, such as histone modifications or non-coding RNA, can be passed on to the next generation through gametes and can affect gene expression in the offspring [13]. In mammals, such an intergenerational inheritance rarely represents a stable transgenerational epigenetic inheritance, but may affect the epigenome reprogramming in the embryo with consequences on embryogenesis and on the health status of the offspring [13].

In this respect, the deep knowledge of epigenetic mechanisms is fundamental to preserving health status and to developing targeted interventions.

The *KISS1* gene was discovered in 1996 as a metastasis suppressor gene in malignant melanoma cells [14], and it was later heavily enrolled in the control of reproduction, with functions related to the sexual differentiation of the brain, the release of hypothalamic gonadotropin-releasing hormone (GnRH), puberty onset, and the maintenance of reproduction in adults [15,16,17,18]. In humans, the *KISS1* gene is located on chromosome 1 (1q32) and encodes a 145 amino acid protein that is proteolytically cleaved in shorter peptides such as kisspeptin-54 (Kp-54), also known as “metastin” for its ability to suppress metastasis, Kp-10, Kp-13, and Kp-14. All kisspeptins (Kps) share a common amidated C-terminal end and are capable of binding and activating the kisspeptin receptor (KISS1R), previously known as GPR54 and originally designed as Hot7t175 or AXOR12 [19,20,21,22]. Recently, a kisspeptin system comprising ligands and receptors was discovered in vertebrates [23]. Apart from cancer-related activities, it has a broader spectrum of actions with direct consequences on gamete quality and fertility rate, pregnancy, energy homeostasis, and body weight control, as recently summarized [24,25].

Due to the multiple facets of kisspeptin activity in biological systems, in this minireview we summarize the epigenetic modulation of the *KISS1* gene in order to provide an example of epigenetic regulation in health and disease.

## 2. The Epigenetic Modulation of *KISS1* in Reproduction

The deep involvement of the kisspeptin system in the central control of reproduction is well known [15], with upcoming data concerning additional peripheral activities [24,25]. Reproduction depends on the physiology of the hypothalamus–pituitary–gonad (HPG) axis. The main actor is the hypothalamic GnRH, which is secreted in a pulsatile manner to target the pituitary gland, thus inducing the secretion of pituitary gonadotropins (follicle-stimulating hormone (FSH) and luteinizing hormone (LH)), the downstream production of sex steroids by gonads, and the progression of gametogenesis [26]. Environmental factors like diet and nutritional status, endocrine disrupting chemicals, stress, or intensive physical training may affect the functionality of the HPG axis with consequences on reproductive ability [15,27,28,29,30,31]. In this respect, several neuronal networks catch and integrate exogenous and endogenous environmental “cues”, thus modulating the activity of GnRH-secreting neurons. Mechanisms depending, among others, on sex steroids or peripheral metabolic biosensors have been suggested [15,26,30], and an inverse relationship between DNA methylation and the *Gnrh1* gene expression during the peripubertal period has been reported [32]. However, in the brain, kisspeptin neurons upstream modulate the secretion of GnRH parallel to permissive or opposing signals mediated by neurokinin B (NKB) and dynorphin (DYN), thus composing the kisspeptin-NKB-DYN neuronal (KNDy) system [15].

In vertebrates, the distribution of kisspeptin neurons in the hypothalamus is sexually dimorphic. In fact, they are mainly located within the arcuate nucleus (ARC) in both males and females and in the rostral periventricular area of the third ventricle (RP3V) of rodents—which contains the sexually dimorphic anteroventral periventricular nucleus (AVPN)—and the anterior preoptic area (POA) of non-rodents in females [15,33]. Such a distribution causes sex-specific changes in *Kiss1* expression and has functional consequences [34]. In fact, the KISS1 neuron population in the ARC is the main target of the negative sex steroid feedback, which occurs in both males and females; the KISS1 neuron population in the AVPN is the main target for estradiol-positive feedback only in females [15]. Interestingly, the expression of both *Kiss1* and *Kiss1r* depends on estradiol [15,23] and a mutual enhancement with estradiol/estradiol receptors (ERs) has been reported [16,35], thus providing evidence that autocrine, paracrine, and endocrocrine pathways affect the endogenous microenvironment and modulate the activity of the kisspeptin system as a consequence.

Several studies, primarily in rodent or cell line models, have investigated the possible epigenetic regulation of *Kiss1* gene in the brain, with a focus on DNA methylation, histone acetylation, and histone methylation [34]. Estrogen responsive element (ERE)-dependent and ERE-independent pathways are responsible for the estradiol-dependent expression of the *Kiss1* gene in the AVPV and ARC, respectively [36]. Epigenetic mechanisms requiring activating histone H3 modification like H3K9/14 acethylation have been discovered [37] and excellently reviewed elsewhere [34,36].

In both animal and human models, the main consequence of kisspeptin signaling impairment is central hypogonadotropic hypogonadism. Conversely, gain-of-function mutations in *KISS1* or *KISS1R* genes cause precocious puberty onset (see [16,38] for recent reviews). As a consequence, the kisspeptin system is currently considered the main gatekeeper of puberty onset, the critical developmental process particularly affected by lifestyle and environmental factors [39,40].

The epigenetic modulation of *Kiss1* or *Kiss1r* genes within the hypothalamus at puberty onset has been investigated in females, providing evidence that the methylation of both *Kiss1* and *Kiss1r* genes promotes changes across puberty [41], with the development of highly significant puberty-specific differential promoter methylation patterns. An epigenetic mechanism of transcriptional repression involving the Polycomb (PcG) silencing complex prevents the premature pubertal process in female rats. In fact, DNA methylation of the PcG genes *Eed* and *Cbx7* precedes puberty, decreasing the expression of both genes. Therefore, the activation of the *Kiss1* gene in the ARC at puberty is the consequence of EED protein loss from the *Kiss1* promoter and activating histone H3 modifications such as H3K4 trimethylation and H3K9/14 acetylation [42]. Consistently, treatment with 5′-Azacytidine (Aza), a well-established DNA methyltransferase (DNMT) inhibitor, from postnatal day 22 to 28 (i.e., juvenile period) caused puberty failure in female rats [42] by means of failed eviction of the EED from the *Kiss1* promoter in the hypothalamus. The epigenetic switch of the *Kiss1* gene from transcriptional repression to activation finds dynamic counterparts in the repression of PcG into mixed-lineage leukemia 1 (MLL1) and 3 (MLL3) [43]. MLL1 and MLL3 are two components of the Trithorax group (TrxG) of modifiers which regulate chromatin remodeling. The first component is capable of changing the chromatin configuration at the promoters of *Kiss1* and *Tac3* from repressive to permissive, which encodes NKB [15]; the second component changes the functional status of a *Kiss1* enhancer from poised to active [43]. However, due to the large number of actors in this physiological process, it is not excluded that the antagonistic epigenetic mechanism of *Kiss1* transcriptional regulation may be common to additional puberty-activating genes like *Nell2*, *TTF1*, etc.

**Figure 1:**
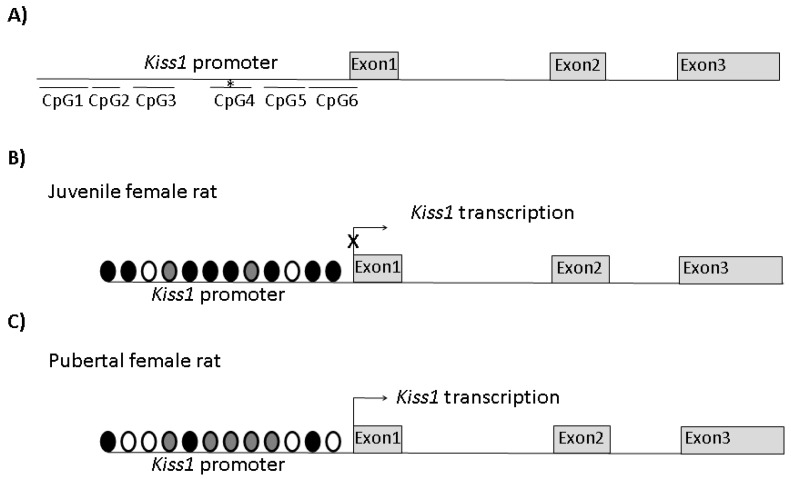
Schematic representation of the main epigenetic changes in the *Kiss1* promoter occurring at puberty in female rats. Six CpG rich regions (CpG1-6) were predicted within the first 2kb 5′ upstream at the transcription start site of *Kiss1*; the methylation status (*) of CpG4 changes at puberty (**A**). The main histone modifications of the *Kiss1* promoter in juvenile (**B**) and pubertal (**C**) rats. Undernutrition and overnutrition mimic conditions in (B) and (C), leading to delayed puberty or precocious puberty, respectively. Black circles, H3K27 trimethylation; white circles, H3K9/14 acethylation; gray circles, H3K4 trimethylation. The length of the *Kiss1* promoter, CpG-rich regions, exons, and introns are not represented in scale. Modified from [41] and [44].

Since puberty onset is highly sensitive to nutritional and metabolic status, the epigenetic effect of diet was recently investigated by Vazquez et al. [44], who designated the sirtuin SIRT1 as fuel-sensing. This NAD^+^-dependent deacethylase was found to be highly expressed within the KISS1 neurons located in the ARC [44]. Interestingly, SIRT1 interacts with the PcG complex and potentiates the repressive activity of the PcG complex on the *Kiss1* promoter by means of a repressive histone configuration on the same promoter, thus contributing to *Kiss1* repression. As for the PcG complex, at puberty SIRT1 is evicted from the promoter of *Kiss1,* leading to the occurrence of *Kiss1* transcription. Both under- and overnutrition exert negative and positive effects, respectively, on puberty by the delayed or premature removal of SIRT1 from the *Kiss1* promoter. As for undernutrition, the central pharmacological activation of SIRT1 or SIRT1 overexpression delays puberty [44]. A schematic representation of the main epigenetic changes of *Kiss1* in a female rat model is reported in Figure 1.

## 3. The Epigenetic Modulation of *KISS1* in Cancer

Cancer is a complex disease characterized by genetic and epigenetic alterations that together contribute to tumor progression. Tumor genome analysis by next-generation sequencing (NGS) highlights the presence of alterations in several epigenetic regulators, suggesting the important role of epigenetic deregulation in cancer development [45]. On these bases, several studies have shown that epigenetic alterations could represent an important target for the use of epigenetic modifiers as therapeutic candidates for some types of cancers [46,47,48].

In tumor development, the epigenome undergoes multiple changes that include hypermethylation in promoter CpG islands—in particular in tumor-suppressor genes—histone modifications that contribute to gene expression alterations, and the deregulation of miRNA expression that is associated with functional changes in target genes [49,50,51,52,53,54,55,56].

*KISS1* was primarily identified as a human malignant melanoma metastasis-suppressor gene [14]. However, later, the involvement of *KISS1/KISS1R* in tumor development was demonstrated in several tumor types [57]. In this respect, the epigenetic regulation of *KISS1* in cancer deserves particular attention, as there is a presently unfulfilled need to identify the alternative pathways required for the expression of the tumor target molecules involved in the development of metastases. In fact, epigenetic drugs have evolved in terms of specificity and efficiency for the treatment of human cancer, representing a potential possibility of successful treatment [46,47,48].

The kisspeptin system has multiple functions in the regulation of tumor progression [57,58,59]. In several cancer types the kisspeptin system has an anti-metastatic role in the regulation of cellular migration and invasion [60]. It might also be involved in other stages of tumor development [58]. For example, *KISS1* is one of the candidate genes involved in the dormancy state, the phase of tumor progression in which patients appear asymptomatic and the disease remains in a state of latency for a variable period of time. In fact, cancer cells could be induced to enter a dormant state to survive within the metastatic niche, causing the metastasis to remain latent for years [61]. *KISS1* expression elicits a dormancy state of the disseminated melanoma cells, inducing a suppression of metastatic colonization to multiple organs [62].

The possible epigenetic modulation of *KISS1* in cancer has been investigated and, although the available data are still relatively few, research in the field is promising for cancer prevention, diagnosis, and treatment. CpG islands are present in the *KISS1* promoter and in cancer the hypermethylation of the *KISS1* promoter results in protein hypoexpression. In colorectal cancer (CRC), epigenetic modifications of the *KISS1* promoter were shown. In particular, hypermethylation of the *KISS1* promoter frequently occurred in CRC samples and rarely in normal tissues. This modification was correlated with transcription and protein expression loss. Therefore, the *KISS1* methylation status was shown to have a diagnostic and prognostic utility for the clinical management of CRC patients. In fact, *KISS1* methylation was related to tumor-grade metastasis, predicted recurrence, and disease-free and overall survival [63]. Therefore, *KISS1* may represent a candidate target for the treatment of metastatic CRC. Furthermore, a combination of the methylation values of *KISS1* and the serum concentration of carcinoembryonic antigen (CEA) have an increased prognostic value in comparison to the evaluation of CEA alone [64].

*KISS1* hypermethylation has also been reported in numerous cases of bladder tumors, in correlation with increasing tumor staging and grading. An epigenetic silencing hypothesis was tested by Cebrian et al. in 2011. These authors related the expression levels of *KISS1* to the histopathological stage of tumors and demonstrated by quantitative reverse transcriptase polymerase chain reaction (RT-PCR) that the methylation of the *KISS1* promoter decreased *KISS1* expression. The low *KISS1* expression alone or in combination with the promoter methylation value was also correlated with poor disease-specific survival. Furthermore, in bladder cancer cells analyzed by methylation-specific PCR and bisulfite sequencing, *KISS1* promoter hypermethylation was frequently reported and related to a low gene expression. [65]. Table 1 summarizes the different studies on tumors showing changes in *KISS1* promoter methylation.

At the molecular level, the epigenetic silencing of *KISS1* in bladder cancer is due to the upregulation of Ubiquitin-like with PHD and RING finger domains 1 (UHRF1). The upregulation of UHRF1 enhances the methylation of CpG nucleotides and downregulates the expression of *KISS1*. UHRF1 was found to be overexpressed in most clinical specimens of bladder cancer in comparison to normal tissues, and in metastatic tumors in comparison to non-metastatic tumors [66].

Lastly, upcoming evidence highlights that miRNAs and long non-coding RNAs (lncRNAs) could modulate Kisspeptin-mediated signaling. Furthermore, miRNAs appear to play an important role in the regulation of proteins that modify and inhibit *KISS1* expression [67]. For example, the expression of *KISS1* is upregulated by the cAMP response element-binding protein (CREB). Additionally, the NAD^+^-dependent de-acetylase SIRT1 prevented the CREB-mediated upregulation of *KISS1* in a mechanism involving *miR-199b.* In fact, *miR-199b* overexpression in CRC represses SIRT1, thus potentiating the CREB-triggered upregulation of *KISS1.* In this respect, *miR-199b* could represent a valid prognostic marker or a new possible therapeutic target for patients with CRC due to its ability to modulate the SIRT1/CREB/KISS1 pathway [68]. However, the exact mechanisms of *KISS1* regulation mediated by non-coding RNAs have not yet been sufficiently outlined, and remain an interesting starting point for future studies.

In any case, the role of *KISS1* in cancer is relevant. However, it is very controversial as a negative or a positive modulator, depending on the cancer context [57]. In several types of tumors, *KISS1* acts as a tumor suppressor gene. Consistently, in pancreatic and ovarian cancer, *KISS1*/*KISS1R* was found to be upregulated in initial phases of cancer development, thus acting as a tumor suppressor. These patients presented a better prognosis and a longer survival rate than those with tumors in which *KISS1* was downregulated by some mechanisms, like promoter hypermethylation (i.e., colorectal and bladder tumors) [57]. Conversely, in triple negative breast cancer (TNBC) cells, which lack estrogen receptor (ER)α, progesterone receptor, and human epidermal growth factor receptor, *KISS1* acts as a tumor promoter, whereas in ERα-positive breast tumors, the situation appears more complex [57]. Hence, recent studies have just pointed out the importance of microenvironment. In fact, it has been demonstrated that *KISS1* and *KISS1R* expression in tumor cells is not sufficient, per se, to predict cancer development behavior [57]. Thus, in the tumor microenvironment, we should evaluate not only the expression of *KISS1*/*KISS1R* in surrounding cells, but also in additional regulation systems such as in the production of cytokines [69,70].

## 4. Conclusions

When observed congruently, both environmental and lifestyle factors induce the epigenetic modulation of the kisspeptin system in physiological and pathological conditions. Thus, the kisspeptin system may represent a possible epigenetic target for the treatment of human diseases and the development of personalized epigenetic therapies in reproduction and cancer.

## Figures and Tables

**Table 1:** Changes in *KISS1* promoter methylation in tumors.

Type of Tumor	Number of Tumors	Methylation (%)	CpG around the Transcription Start Site	References
Colorectal cancer (CRC)	126	83.3	-	[63]
CRC	352	72.7	19	[64]
Bladder cancer	804	83.1	19	[65]
